# Brown Algae *Dictyopteris divaricata* Attenuates Adipogenesis by Modulating Adipocyte Differentiation and Promoting Lipolysis through Heme Oxygenase-1 Activation in 3T3-L1 Cells

**DOI:** 10.3390/md22020091

**Published:** 2024-02-16

**Authors:** Lakshi A. Dayarathne, Seok-Chun Ko, Mi-Jin Yim, Jeong Min Lee, Ji-Yul Kim, Gun-Woo Oh, Chul Hwan Kim, Kyung Woo Kim, Dae-Sung Lee, Jae-Young Je

**Affiliations:** 1 Department of Food and Nutrition, Pukyong National University, Busan 48513, Republic of Korea; lakshid31@gmail.com; 2 National Marine Biodiversity of Korea (MABIK), Seochun 33662, Republic of Korea; seokchunk@mabik.re.kr (S.-C.K.); miyim@mabik.re.kr (M.-J.Y.); ljm06@mabik.re.kr (J.M.L.); jiyul2224@mabik.re.kr (J.-Y.K.); ogwchobo@mabik.re.kr (G.-W.O.); kchulh1204@mabik.re.kr (C.H.K.); kimkw79@mabik.re.kr (K.W.K.); daesung@mabik.re.kr (D.-S.L.); 3 Major of Human Bioconvergence, Division of Smart Healthcare, Pukyong National University, Busan 48513, Republic of Korea

**Keywords:** *Dictyopteris divaricata*, adipogenesis, lipid accumulation, lipolysis, HO-1

## Abstract

The present study aims to explore the probable anti-adipogenesis effect of *Dictyopteris divaricata* (*D. divaricata*) in 3T3-L1 preadipocytes by regulating heme oxygenase-1 (HO-1). The extract of *D. divaricata* retarded lipid accretion and decreased triglyceride (TG) content in 3T3-L1 adipocytes but increased free glycerol levels. Treatment with the extract inhibited lipogenesis by inhibiting protein expressions of fatty acid synthase (FAS) and lipoprotein lipase (LPL), whereas lipolysis increased by activating phosphorylation of hormone-sensitive lipase (p-HSL) and AMP-activated protein kinase (p-AMPK). The extract inhibited adipocyte differentiation of 3T3-L1 preadipocytes through down-regulating adipogenic transcription factors, including peroxisome proliferator-activated receptor gamma (PPARγ) and CCAAT/enhancer-binding protein α (C/EBPα), and sterol regulatory element-binding protein 1 (SREBP1). This is attributed to the triggering of Wnt/β-catenin signaling. In addition, this study found that treatment with the extract activated HO-1 expression. Pharmacological approaches revealed that treatment with Zinc Protoporphyrin (ZnPP), an HO-1 inhibitor, resulted in an increase in lipid accumulation and a decrease in free glycerol levels. Finally, three adipogenic transcription factors, such as PPARγ, C/EBPα, and SREBP1, restored their expression in the presence of ZnPP. Analysis of chemical constituents revealed that the extract of *D. divaricata* is rich in 1,4-benzenediol, 7-tetradecenal, fucosterol, and n-hexadecanoic acid, which are known to have multiple pharmacological properties.

## 1. Introduction

A chronic metabolic syndrome, obesity, is linked with numerous metabolic complications including cardiovascular illnesses, hypertension, and type 2 diabetes [1]. The key cause is the imbalance in energy input and output which leads to a rise in adipocyte number and larger adipocyte size due to the accumulation of body fats via adipogenesis [2]. Adipogenesis is known to be a well-orchestrated multifarious process that involves the development of preadipocytes that resemble fibroblast to mature adipocytes via serial activation of several transcription factors namely CCAAT/enhancer-binding protein (C/EBPα), peroxisome proliferator-activated receptor-γ (PPAR-γ), and sterol regulatory element-binding protein-1 (SREBP-1) [3]. Furthermore, the exhibition of subsequent target genes, which entails lipid synthesis and lipolysis is primarily modulated by these factors [4]. 

Among the isoforms of Heme oxygenase (HO), HO-1 is a highly inducible molecule produced as a result of oxidative stress and it degrades heme to biliverdin followed by synchronous delivery of CO and iron which have crucial regulatory functions in cells. According to recent developments in adipogenic research, increased HO-1 levels in obesity cause the enlarged adipocytes to shrink, which lowers the reduction in visceral and subcutaneous fat content [5]. In addition, inducing HO-1 activity is a conceivable path for the increment occurrence of metabolic syndrome, an outbreak of obesity, dyslipidemia, hypertension, and diabetes [6]. Thus, pharmaceuticals that enhance HO-1 activity have been utilized as a therapeutic tactic to treat obesity and related complications [7]. Accumulative pieces of evidence have revealed that natural products including peptides, polyphenols, and polysaccharides showed anti-adipogenesis effects in various research models through HO-1 induction [8,9,10].

Over the last decade, the significance of bioactive offshoots as functional constituents has been exhibited by their favorable health outcomes. Thus, the detailed identification of new bioactive constituents, with biological ventures from marine algae, has drawn increased concern in the pharmacology field compared with marine sources [11]. Innumerable amounts of carotenoids, fatty acids, peptides, phenols, and minerals can be found in abundance in marine algae [12]. Since early times, seaweeds have appeared in the holistic medicine and cuisine of most Asian countries; for instance, South Korea, China, and Japan [13]. Epidemiological studies have exhibited a correlation between seaweed consumption and the decreased occurrence of chronic illnesses such as cardiovascular complications, cancers, and hyperlipidemia [14]. While research in this field is ongoing, several marine compounds have shown promising anti-adipogenesis potential. Several studies showed that bioactive compounds isolated from marine algae impede adipocyte differentiation with reduced lipid levels in differentiated 3T3-L1 cells [15,16]. Furthermore, rats fed a high-fat diet along with seaweed powder have reduced body weight gain and lower plasma levels in cholesterol and triacylglycerols [17,18]. *Dictyopteris divaricata* (*D. divaricata*) a brown algae from *Dictyotaceae* family has been utilized as a rich source of fortified food and remedial agent to date due to its various functional compounds [19]. For *D. divaricata,* multiple functions of therapeutic concerns have been elucidated. The ethanolic extract of *D. divaricata* has shown anti-cancer effect in eukaryotic cell lines and dichloromethane/methanol and ethanol extracts are known for α-Glucosidase inhibition activity which might be potential remedy for diabetes [20,21,22]. Furthermore, polysaccharides extracted from *D. divaricata* have exhibited potent anti-oxidant effects and possess immune-stimulatory activities [19]. Thus, suggesting *D. divaricata* as a functional food to improve human health. However, there is no previous work reporting on the anti-adipogenesis potential of *D. divaricata* underlying its mechanism of action and this remains to be explored. The physical and chemical properties of a natural extract is strongly influenced by the extraction method, emphasizing the extraction method has a strong impact on its pharmacological efficiency [23]. The ethanolic extraction of natural compounds has been widely used in pharmaceutical formulations due to high solubility and low toxicity. This is crucial when considering potential human applications. A number of previous studies reported that ethanolic extracts of marine algae and other marine sources show strong therapeutic effects over the other extraction methods [24,25,26,27]. Therefore, the present study investigates the effect of the hydroethanolic extract of *D. divaricata* on adipogenesis and its molecular interaction of action, with a prime core of HO-1 regulation by *D. divaricata* in 3T3-L1 adipocytes.

## 2. Results

### 2.1. Characterization of Chemical Constituents in D. divaricata

**Figure 1:**
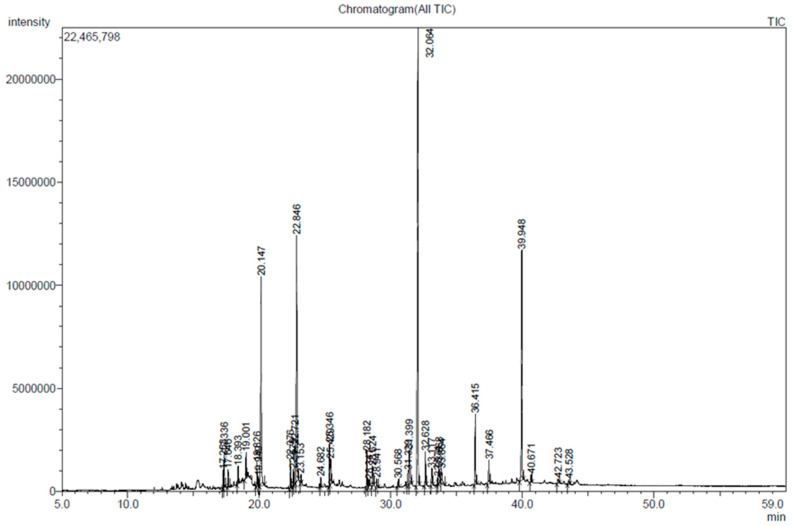
GC-MS chromatogram of *D. divaricata* extract.

The total flavonoid content (TFC) of the *D. divaricata* extract was 19.98 ± 0.7 mg rutin equivalent (RE)/g. The total phenolic content (TPC) of the *D. divaricata* extract was 6.03± 0.9 mg galic acid equivalent (GAE)/g. The Gas chromatography–mass spectrometry (GC-MS) chromatogram and detected compounds of the *D. divaricata* extract are given in Figure 1 and Table 1. In a comparison of the mass spectra of the compounds with the National Institute Standard and Technology (NIST) library, 20 peaks were acquired and the phytoconstituents were characterized and identified with retention times and relative percentages. Based on the peak area the major metabolite identified was 1,4-benzenediol,2-decahydro-5,5,8a-trimethyl-2-methylene-1-(Zonarol) (28.59%), followed by 7-tetradecenal (14.79%), fucosterol (12.45%), and n-hexadecanoic acid (11.46%). Additionally, GC-MS analysis confirmed the presence of several saturated and unsaturated fatty acids including tetradecanoic acid, hexadecenoic acid, linoelaidic acid, arachidonic acid, and octadecanoic acid in the algae extract. Furthermore, several fatty alcohols and carotenoid metabolites (-)-loliolide were also reported in the extract. Taken together, the GC-MS results suggested that these different major compounds in *D. divaricata* extract might be the active compounds that cause the anti-adipogenesis effect in 3T3-L1 adipocytes.

### 2.2. Effect of D. divaricata on Cell Viability

**Figure 2:**
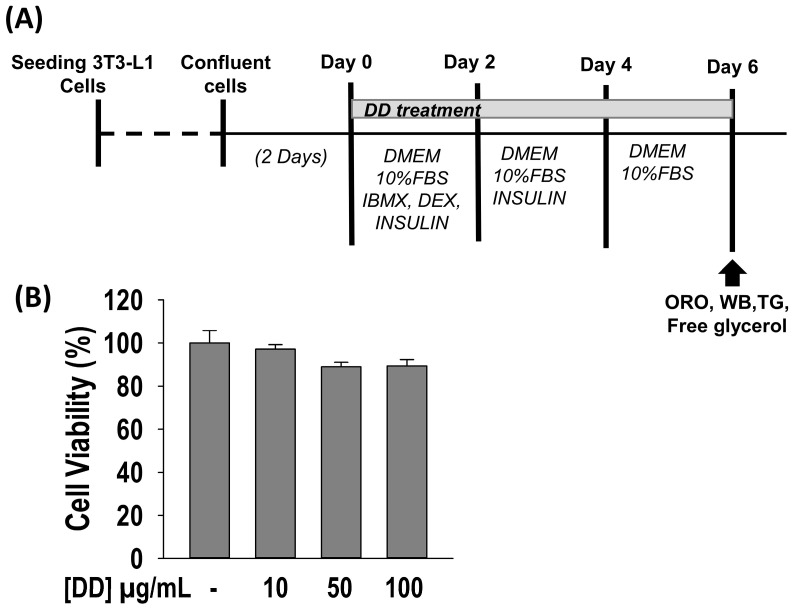
Effect of *Dictyopteris divaricata* (DD) on viability of 3T3-L1 cell. (**A**) Differentiation procedure and assay schedule; (**B**) Effect of DD on 3T3-L1 cell viability. The cells were incubated with 10–100 µg/mL of DD for 48 h, and cell viability was assessed by MTT assay. All data given are means ± SD (n = 3) and error bars with different letters are significantly different at *p <* 0.05. DMEM, Dulbecco’s modified Eagle’s medium; DEX, dexamathanose; FBS, fetal bovine serum; IBMX, 3-isobutyl-1-methylxanthine; INS, insulin; MTT, 3-(4,5-dimethylthiazol-2-yl)-2,5-diphenyltetrazolium bromide; ORO, Oil red O; TG, triglyceride; WB; Western blot.

The differentiation assay schedule of 3T3-L1 cells is shown in Figure 2A. 3-(4,5-dimethylthiazol-2-yl)-2,5-diphenyltetrazolium (MTT) assay was carried out at the outset to ensure the cytotoxicity of the sample. Cellular cytotoxicity was not observed for up to 100 µg/mL (Figure 2B). Accordingly, further assays were performed at non-toxic concentrations of the sample.

### 2.3. D. divaricata Inhibits Lipid Accumulation and TG Content, and Promotes Free Glycerol Release

**Figure 3:**
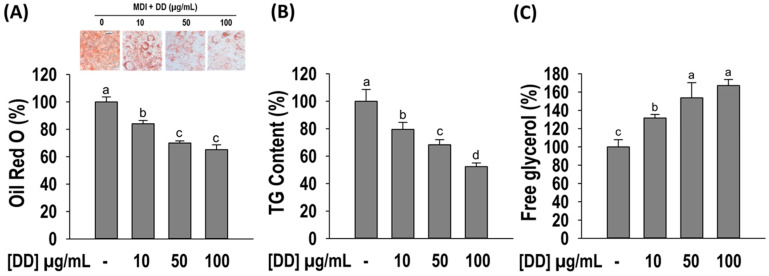
Effect of *Dictyopteris divaricata* (DD) on adipogenesis of 3T3-L1 cells. (**A**) Illustrative images and quantitative analysis of lipid accumulation; (**B**) intra-cellular TG accumulation; (**C**) free glycerol release. Cells were differentiated in the presence and absence of DD for 6 days as described in material and methods followed by staining with Oil Red O reagent, colorimetric determination of TG content, and free glycerol release assay. All data given are means ± SD (n = 3) and error bars with different letters are significantly different at *p <* 0.05. MDI, differentiation initiation media; TG, triglyceride.

The effect of *D. divaricata* on lipid accumulation and TG content was assessed by Oil Red O staining and TG assay (Figure 3A,B). The differentiated control adipocytes showed a significant accumulation of intra-cellular lipids and TG content. As opposed to the control, the sample-treated adipocytes decrease the lipid accumulation and TG content significantly (*p* < 0.05). *D. divaricata* at 100 µg/mL concentration reduces the lipid accumulation and TG content by 60% and 50%, respectively. To assess the lipolysis effect of *D. divaricata*, free glycerol release by the 3T3-L1 adipocytes was quantified by performing the free glycerol release assay. After 6 days of sample treatment, high amounts of free glycerol were significantly released compared to the differentiated cells (Figure 3C), indicating that *D. divaricata* induced lipolysis in matured 3T3-L1 adipocytes. The above results reveal that *D. divaricata* extract inhibits adipogenesis through reduced lipid accumulation and TG content, and promotes lipolysis by 3T3-L1 adipocytes.

### 2.4. D. divaricata Inhibits Expression of Key Adipogenic Markers and Stimulates β-Catenin Nuclear Translocation 

**Figure 4:**
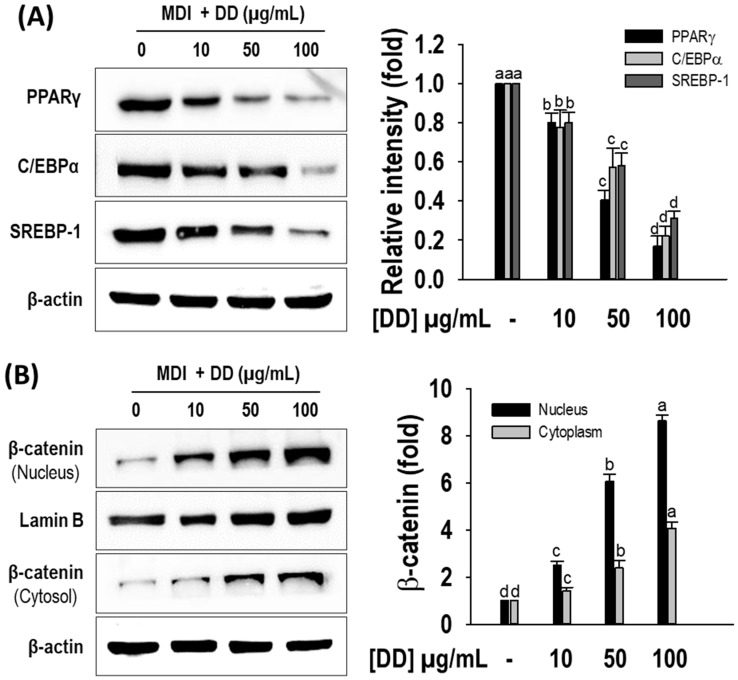
Effect of *Dictyopteris divaricata* (DD) on protein levels of PPARγ, C/EBPα, SREBP-1, and β-catenin by 3T3-L1 cells. (**A**) Representative Western blots of PPARγ, C/EBPα, and SREBP-1; relative intensities were normalized to the band of beta-actin; (**B**) Representative Western blots of β-catenin; relative intensities of nucleus and cytosol fractions were normalized to the band of Lamin B and β-actin, respectively. Cells were differentiated in the presence and absence of DD for 6 days as described in materials and methods, followed by Western blot analysis. All data given are means ± SD (n = 3) and error bars with different letters are significantly different at *p <* 0.05. MDI, differentiation initiation media; PPARγ, peroxisome proliferator-activated receptor gamma; C-EBPα, CCAAT/enhancer-binding protein alfa; SREBP-1, sterol regulatory element-binding protein-1.

Furthermore, to clarify the underlying molecular mechanism, Western blotting was performed to observe the effect of *D. divaricata* on the expression of principal adipogenic transcription factors namely PPARγ, C/EBPα, and SREBP-1. As appears in Figure 4A, the protein levels of PPARγ, C/EBPα, and SREBP-1 transcription factors were significantly reduced (*p* < 0.05) by the sample treatment compared to the differentiated control. Furthermore, the β-catenin levels in cytosolic and nuclear fractions were observed by Western blotting. Figure 4B shows that *D. divaricata* (10–100 µg/mL) up-regulated β-catenin expression in matured 3T3-L1 adipocytes compared to untreated adipocytes in both the cytosolic and the nuclear fraction. Furthermore, the fold change in the β-catenin level in the nucleus fraction was comparatively higher than the cytosolic fraction, demonstrating the nuclear β-catenin translocation. The results indicated the reduced lipid accumulation by *D. divaricata* was mediated via down-regulating PPARγ, C/EBPα, and SREBP-1 expressions and up-regulating β-catenin expression.

### 2.5. D. divaricata Regulates Lipolysis and Lipogenesis Target Protein Expression 

**Figure 5:**
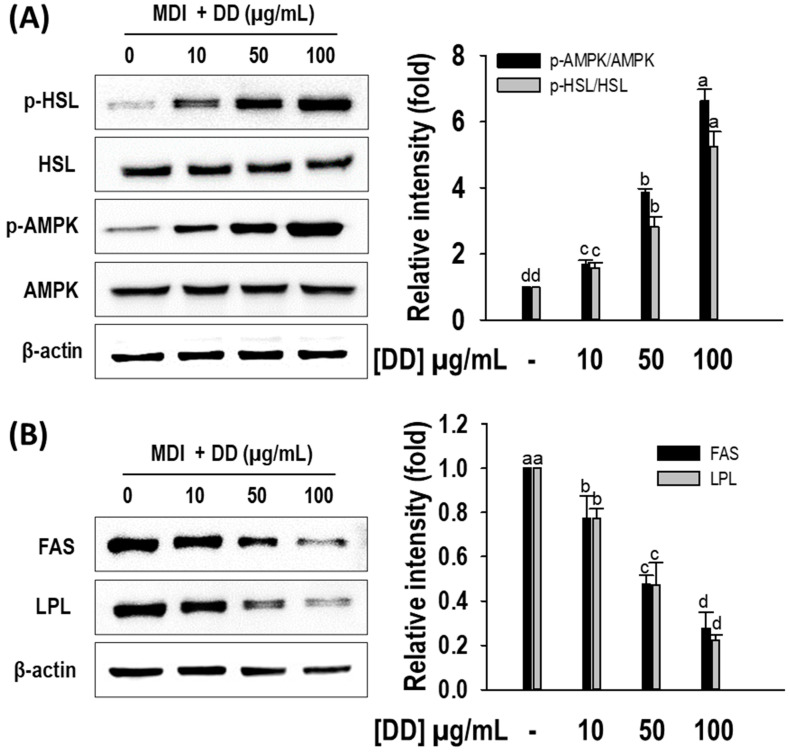
Effect of *Dictyopteris divaricata* (DD) on protein levels of AMPK, HSL, FAS, and LPL by 3T3-L1 cells. (**A**) Representative Western blots of p-HSL and p-AMPK; relative intensities were normalized to the band of HSL and AMPK, respectively; (**B**) Representative Western blots of FAS and LPL; relative intensities were normalized to the band of β-actin. Cells were differentiated in the presence and absence of DD for 6 days as described in the materials and methods section, followed by Western blot analysis. All data given are means ± S. D (n = 3) and error bars with different letters are significantly different at *p <* 0.05. MDI, differentiation initiation media; p-HSL, phosphorylation of hormone-sensitive lipase; p-AMPK, phosphorylation of AMP-activated protein kinase; FAS, fatty acid synthase; LPL, lipoprotein lipase.

To confirm the lipolysis effect of *D. divaricata*, the phosphorylation of hormone-sensitive lipase (HSL) and AMP-activated protein kinase (AMPK) was observed by performing Western blotting. *D. divaricata* stimulated the phosphorylation of HSL and AMPK, indicating that *D. divaricata* enhanced lipolysis (Figure 5A). In addition, the extract degraded the protein levels of fatty acid synthase (FAS) and lipoprotein lipase (LPL) compared to the control, thereby inhibiting lipogenesis (Figure 5B). The results suggest that the presence of *D. divaricata* during the adipogenesis process induced the up-regulation of the lipolysis target protein and down-regulated lipogenesis target proteins dose-dependently.

### 2.6. D. divaricata Promotes HO-1 Activation

**Figure 6:**
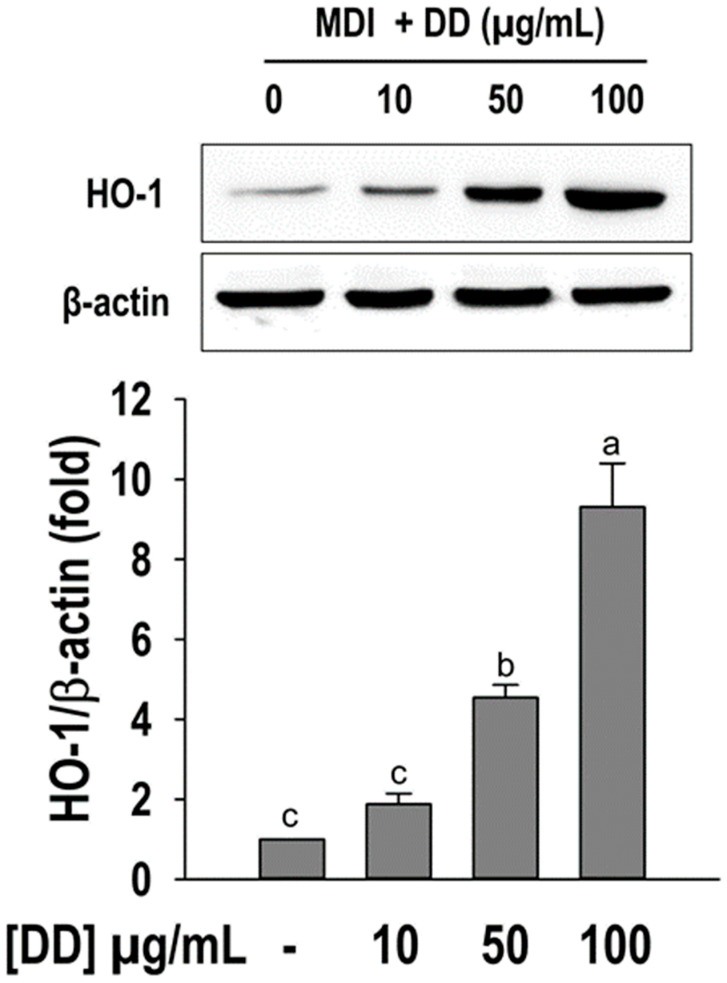
Effect of *Dictyopteris divaricata* (DD) on HO-1 activation. Representative Western blot of HO-1. The relative intensity of HO-1 was normalized to the band of β-actin. Cells were differentiated in the presence and absence of DD for 6 days as described in materials and methods followed by Western blot analysis. All data given are means ± SD (n = 3) and error bars with different letters are significantly different at *p <* 0.05. MDI, differentiation initiation media; HO-1, heme oxygenase 1.

To observe whether the *D. divaricata* extract induce the HO-1 induction its activation was determined via Western blotting. In compared with differentiated control the treatment of *D. divaricata* significantly induced (*p* < 0.05) the HO-1 protein levels and it was dose-dependent (Figure 6). *D. divaricata* at 100 µg/mL concentration showed a nearly 10-fold increase in HO-1 protein level compared to the control.

### 2.7. ZnPP Effect on D. divaricata Mediated Anti-Adipogenic Effect 

**Figure 7:**
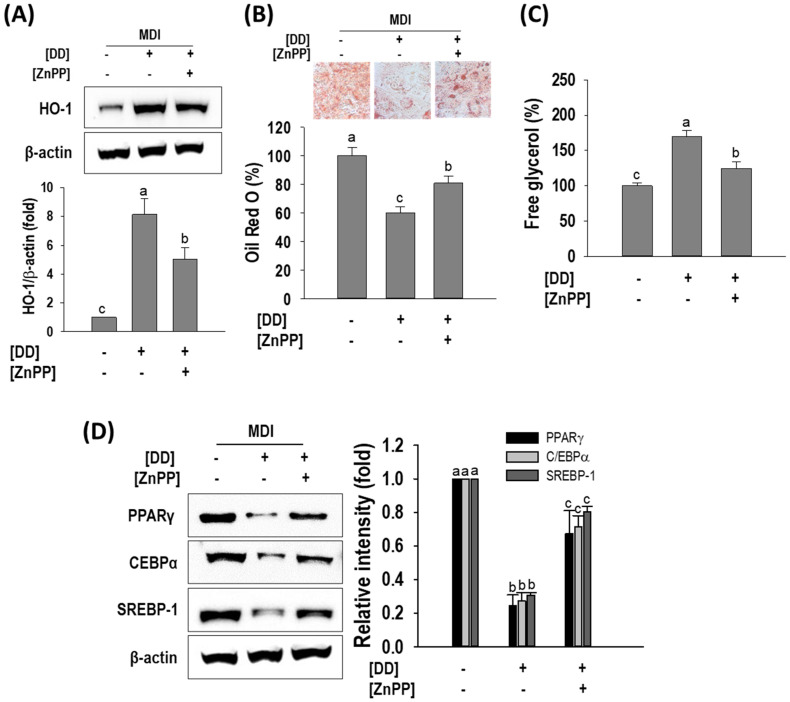
*Dictyopteris divaricata* (DD) induced HO-1 activation and HO-1 inhibitor attenuates adipogenesis inhibition by DD. (**A**) Representative Western blot of HO-1. (**B**) Illustrative images and quantitative analysis of lipid accumulation; (**C**) Free glycerol release; (**D**) Representative Western blots C/EBPα, PPARγ, and SREBP-1; relative intensities were normalized to the band of β-actin. Cells were differentiated and treated with DD as described in materials and methods with 2 h pretreatment of ZnPP (10 µM) followed by Western blot analysis, Oil Red O staining, and free glycerol release. All data given are means ± SD (n = 3) and error bars with different letters are significantly different at *p* < 0.05. MDI, differentiation initiation media; HO-1, heme oxygenase 1. PPARγ, peroxisome proliferator-activated receptor gamma; C-EBPα, CCAAT/enhancer-binding protein alfa; SREBP-1, sterol regulatory element-binding protein-1.

To further confirm the role of HO-1 in the anti-adipogenic effect of *D. divaricata*, its function was particularly suppressed with Zinc Protoporphyrin (ZnPP), and the level of lipid accumulation, TG content, and free glycerol release was assessed. Furthermore, the expression of three main adipogenic factors was observed via Western blotting. As appeared in Figure 7A, ZnPP exposure has reduced the HO-1 induction by *D. divaricata*. The level of the reduced lipid accumulation by *D. divaricata* was also increased by ZnPP pretreatment (Figure 7B). Whereas, the increased free glycerol by *D. divaricata* was diminished by ZnPP pretreatment (Figure 7C). Finally, this study investigated the PPARγ, C/EBPα, and SREBP-1 protein levels in the existence of ZnPP. As shown in Figure 7D, the down-regulated protein levels of PPARγ, C/EBPα, and SREBP-1 by *D. divaricata* were partially restored by ZnPP pretreatment, indicating that HO-1 plays a crucial event in the regulation of adipocyte differentiation.

## 3. Discussion

It is widely known that the development of obesity is correlated with adipocyte differentiation and accumulation of fat. Possible approaches for alleviating obesity include either reducing the assimilation of free fatty acid to triglycerides or enhancing lipolysis [28]. Thus, the current study investigates the effect of *D. divaricata* on adipogenesis by assessing lipid accumulation, TG content, and the expression of adipogenesis and lipolysis proteins in 3T3-L1 adipocytes. 

The transcriptional activators including C/EBPα, PPARγ, and SREBP-1 play a pivotal function in adipogenesis. C/EBPα and PPARγ function by coordinating the gene expression that is involved in the establishment of mature adipocytes. Cumulative evidence showed that β-catenin is a negative adipogenesis mediator and it is positively linked with the decreased function of PPARγ via a functional interplay between the catenin-binding domain of PPARγ and the T-cell factor/lymphoid enhancer factor (TCF/LEF)-binding domain of β-catenin. Moreover, β-catenin triggers the transcription of downstream target genes, for instance, Cyclin D1 (CCND1), which represses the function of PPARγ and C/EBPα [29,30,31]. Furthermore, SREBP-1 is the principal regulator of fatty acid assimilation and plays a central event in the transcriptional regulation of numerous lipogenic genes that mediate lipid synthesis [32]. The stimulation of C/EBPα and PPARγ together with SREBP-1 leads to final differentiation through their subsequent transcription of adipocyte-specified genes namely LPL and FAS [33]. FAS demonstrates a pivotal function in the long-term control of lipid biosynthesis. It stimulates the palmitate de novo synthesis out of acetyl CoA and maloinyl CoA and also assists in the production and aggregation of triglyceride in adipocytes [34]. LPL catalyzes the hydrolysis of triglycerides, playing a key role in the efficient uptake and storage of fatty acid in cells; therefore, regulation of LPL function in adipocytes is intently connected to obesity [35]. Therefore, repressing the expression of adipogenic biomarkers is an efficient approach for inhibiting adipocyte differentiation and lipid accumulation in 3T3-L1 adipocytes. In the current study, the *D. divaricata* extract notably reduced the Oil Red O staining levels with significantly reduced amounts of accumulated intracellular TG suggesting that *D. divaricata* suppressed the conversion of preadipocytes into mature adipocytes and also reduced the assimilation of lipid droplets. In addition, the presence of *D. divaricata* down-regulated PPARγ, C/EBPα, and SREBP-1 with increased nuclear localization of β-catenin and further down-regulated the protein levels of FAS and LPL, which suggested that *D. divaricata* suppressed adipogenesis though PPARγ and C/EBPα mediated mechanism. Consistent with the present findings, several other studies reported that suppressing the expression of adipogenic-related proteins positively modulates the adipogenesis in 3T3-L1 cells. For instance, Lee et al. reported that ethanolic extract of red seaweed showed strong inhibition of lipid accumulation in 3T3-L1 adipocytes by down-regulating the adipogenic proteins SREBP-1, PPAR-γ, and C/EBP-α [27]. Furthermore, several studies showed that brown algae possess an anti-adipogenesis effect by mediating the down-regulation of adipogenesis-related proteins [36,37,38].

Energy homeostasis is a fundamental aspect of living organisms, which provides a stable equilibrium between fat deposition and mobilization [39]. TG in adipocytes is rapidly mobilized through lipolysis when there is an energy deficit, by hydrolyzing TG into free fatty acids and glycerol; thus, the stimulation of lipolysis is a favorable approach for obesity treatment. Hence, we hypothesized whether the reduced adipogenesis by *D. divaricata* extract is associated with lipolysis. As expected, the extract treatment for 6 days stimulates the free glycerol release by 3T3-L1 cells. The adipocyte’s lipolysis is controlled by HSL which catalyzes the hydrolysis of diacylglycerol, the key rate-determining step in adipocytes during lipolysis [40,41]. AMPK is the master regulator of lipid metabolism which involves adipogenesis, lipolysis, and fatty acid β-oxidation in adipocytes. AMPK activation phosphorylates HSL which accelerates lipolysis [42,43]. In addition, it suppresses adipocyte differentiation by directly down-regulating the key adipogenic transcription factors [44]. Therefore, the protein expressions of HSL and AMPK were observed via Western blotting. The findings reveal that *D. divaricata* increased lipolysis during the formation of mature adipocytes from the preadipocytes by stimulating the phosphorylation of HSL and AMPK. In accordance with the present findings, previous studies also reported that brown algae are composed of several active compounds that induce lipolysis with increased expressions of HSL and AMPK [32,45,46].

HO-1 is an intuitive enzyme which involved in the rate-limiting stage of free heme breakdown to produce biliverdin, carbon monoxide, and iron. The decreased expression of HO-1 and its function leads to elevated cellular heme levels that promote adipocyte differentiation [47]. The latest developments in obesity studies reported that elevated HO-1 levels in obese mice suppressed adipogenesis with an increased number of small adipocyte content and decreased quantity of enlarged adipocytes [48]. In addition, several studies showed that induced HO-1 expression has a direct involvement in regulating adipogenic markers. Furthermore, the deprivation of HO-1 activity by siRNA silencing of HO-1 resulted in increased expressions of C/EBPα and PPARγ proteins in addition to an increment in the commencing of adipocyte differentiation from mesenchymal stem cells in vitro [49]. Thus, enhanced HO-1 expression has proven to be a key determinant in regulating adiposity. The present study demonstrated that *D. divaricata* has increased the HO-1 abundance in 3T3-L1 preadipocytes. Since increased HO-1 expression demonstrates a crucial role in adipogenesis, in this study, HO-1 antagonist, ZnPP was used to validate the role of HO-1 induction by *D. divaricata* treatment in adipogenesis. As anticipated, the expression level of HO-1 over the time of adipocyte differentiation with the presence of *D. divaricata* decreased with ZnPP treatment. Consequently, the suppression of PPARγ, C/EBPα, and SREBP-1 by *D. divaricata* were retrieved through ZnPP treatment. Furthermore, this study demonstrated that ZnPP treatment reversed lipolysis and restored lipogenesis. Taken together the current study results confirmed that HO-1 activation by *D. divaricata* is a principal outcome in anti-adipogenesis in 3T3-L1 adipocytes. We believe that this is the first study that shows a direct correlation of HO-1’s role in inhibiting adipogenesis by marine brown algae. However, a study performed by Eo et al. showed that *Ecklonia cava* (a brown algae) polyphenol extract has favorable effects on hepatic lipid metabolism, inflammation, and oxidative stress together with increased HO-1 levels [50]. Furthermore, fucoxanthin, which was isolated first from brown algae, showed anti-oxidant effects with up-regulation of HO-1 [51]. Despite that, a previous study showed that HO-1 activation by blue muscle hydrolysates inhibits adipogenesis through HO-1/Nrf2 up-regulation in mesenchymal stem cells [8]. Apart from marine origin, several other naturally occurring compounds are known to reduce adipogenesis via HO-1 up-regulation [1,52,53]. Seo et al. reported that HO-1 activation by pterostilbene, a naturally available phenolic compound in blueberries, controls early-stage adipogenic markers which arrests further progression of adipogenesis in 3T3-L1 cells [54]. Taken together, it can be suggested that HO-1 activation by functional bioactives has a significant impact on ameliorating obesity and associated disorders.

Marine algae often live-in extreme environments of light, salinity, and temperature. To withstand in this environment, these algae produce a diverse array of functional metabolites. These functional compounds comprise numerous lipids, carotenoids, polyphenols, and some polysaccharides that are well aware of their health-nurturing attributes [55,56]. Recently, the trending demand for marine algae as a phytonutrient resource has increased their usage in the field of nutraceuticals due to their various beneficial features including anti-adipogenic, anti-oxidant, and anti-diabetic properties [57,58,59]. According to the GC-MS analysis, *D. divaricata,* used in the present study, contained Zonarol, 7-tetradecenal, fucosterol, and n-hexadecenoic acid in a majority together with several fatty acids, amides, alcohols and carotenoid metabolites. The presence of these compounds in *D. divaricata* was similar to previous studies [60,61]. Marine algal lipids are likely to have high functionalities as they are enriched with omega-6 and omega−3 fatty acids, including polyphenols, fucosterols, and phospholipids, to name a few [62]. A study on brown algae demonstrated the algal lipids from *Padina tetrastromatica* as a functional component to attenuate adipocyte development and promote thermogenesis in diet-induced obesity C57BL6 mice [63]. It has been reported that fucosterol—a sterol element mainly procured from brown algae—retards the adipocyte differentiation via C/EBPα and PPARγ-mediated adipogenesis mechanism [2,64]. In addition, phenolic compound (−)-loliolide isolated from *Sargassum horneri* has demonstrated relatively low levels of lipid accumulation via modulation of adipogenesis, lipolysis, and thermogenesis-related proteins in 3T3-L1 adipocytes [46]. Furthermore, a previous study has reported that the administration of Zonarol-rich extract from brown algae exhibits a modulatory role in the lipid metabolism of liver cells, which leads to reduced lipid accumulation and improved lipid metabolism by down-regulating SREBPs and PPARγ [65]. Despite the improved lipid metabolism potential of Zonarol, a previous study demonstrates that Zonarol and the active compound of crude extract of *Dictyopteris undulata* exhibit a neuroprotective effect against oxidative stress in HT22 hippocampal neuronal cells with a notable increase in HO-1 protein abundance and activating the Nrf2/ARE pathway [66]. Taken together, the anti-adipogenesis effect of *D. divaricata* is probably due to the presence of individual compounds or combinations of more than one compound.

## 4. Materials and Methods

### 4.1. Materials

3T3-L1 cells were procured from the American Type Culture Collection (ATCC, Manassas, VA, USA). Every other reagent utilized in the study was obtained from Sigma-Aldrich unless otherwise noted (St. Louis, MO, USA). All materials for cell culture were purchased through Life Technologies (Gibco BRL, Grand Island, NY, USA). All antibodies are products from Santa Cruz Biotechnology (Santa Cruz, CA, USA).

### 4.2. Preparation of D. divaricata Extract

The *D. divaricata* extract used in the study was kindly gifted by the National Marine Biodiversity Institute of Korea (MABIK, Seochun, Republic of Korea). The brown alga, *D. divaricata,* was harvested in Gangneung, Gangwon state, Republic of Korea. A voucher specimen (NP-0112) was placed at MABIK. After washing *D. divaricata* with tap water, it was stored at −80 °C. Then the frozen samples were subjected to lyophilization and homogenization by a grinder. The dried powder (250 g) was wrenched out with 2.5 L of 70% (*v*/*v*) EtOH (1:10 *w*/*v*) for 1 h (five repeats) by sonication and the dried extract was obtained by Vacuo evaporation. The resulted dried extract of 3.67 g (1.47%) was deposited at −80 °C until the biological effect assessment.

### 4.3. Total Flavonoid Content (TFC) and Total Phenol Content (TPC)

For TFC and TPC, 20 mg of sample was used. The TFC of each extract was quantified according to a previously established method [67]. Briefly, the extract was mixed with distilled water and 5% NaNO_2_ was added and incubated for 5 min at room temperature. After adding 10% AlCl_3_ the mixture of solution was incubated for 50 min at room temperature. At last, the absorbance was recorded at 510 nm after adding 1 M NaOH. The calibration curve was obtained from the rutin solution as the reference compound. TFC was denoted as mg rutin equivalents per gram of dried extract (mg RE/g). 

TPC present in algal extract was quantified by the Folin-Ciocalteu method described earlier [68]. Briefly, the extract was mixed with diluted Folin-Ciocalteu solution followed by 7.5% Na_2_CO_3_ (80 µL). After incubation at room temperature for 2 h, absorbance was taken at 600 nm. The calibration curve was obtained from gallic acid as the reference. The TPC amount was denoted as mg gallic acid equivalents per gram of dried extract (mg GAE/g).

### 4.4. Characterization of Active Compounds 

A Shimadzu QP-2010 Ultra GC-MS (Shimdasu, Kyoto, Japan) equipped with an Agilent DB-5MS UI column (30 m × 0.25 mm × 0.25 μm) with helium at 1.0 mL/min as the carrier gas was used for GC-MS analysis. 10 mg of sample was dissolved in 1 mL of methanol and injected at 2 μL. The splitless mode was followed for the injection with the split ratio of 50:1, and the injection temperature was set at 280 °C. The oven temperature was initially programmed at 60 °C, held at that temperature for 2 min after injection and was raised to 200 °C at a rate of 10 °C/min, and subsequently raised to 320 °C at a ramp rate of 5 °C/min and held for 20 min. The ion source and interface temperature were set at 200 °C and 250 °C, correspondingly. Mass spectra were recorded under electron impact ionization at 70 eV of electron energy with the scanning range of *m*/*z* 40–600. The spectra of the unknown components of the *D. divaricta* fraction obtained were identified based on their retention indices and interpretation of mass spectrum was achieved by comparison to the authentic standard mass spectra of known components stored in the National Institute Standard and Technology (NIST), NIST V 11 data library with more than 62,000 patterns. 

### 4.5. Cell Culture

3T3-L1 fibroblasts were cultured in Dulbecco’s modified Eagle’s medium (DMEM) with 10% bovine serum; 1% penicillin/streptomycin (PS) in a humidified atmosphere containing 5% CO_2_ at 37 °C.

### 4.6. Induction of Adipocyte Differentiation

The 3T3-L1 fibroblasts were maintained in 48-well plates (3 × 10^4^ cells/well) and 6-well plates (3 × 10^5^ cells/well), and 48 h following post confluence, differentiation of the cells were induced using Differentiation Initiation Media (MDI) containing DMEM, 1% PS, 10% fetal bovine serum (FBS) 0.5 mM 3-Isobutyl-1-methylxanthine (IBMX), 0.5 μM dexamethasone (DEX) and 10 μg/mL insulin for 48 h. In due course, the MDI was replaced with a routine medium (DMEM with 1% PS and 10% FBS) supplemented with 10 μg/mL insulin. After 48 h of incubation, media were replenished to normal culture media every 48 h until completion of the differentiation. To assess the effects of the *D. divaricata* extract on adipogenesis, cells were cultured in MDI with or without the extract until the completion of the differentiation of adipocytes. Matured adipocytes at day 6 were used for further assays.

### 4.7. Cell Cytotoxicity Evaluation

The 3T3-L1 fibroblasts were cultured at the cell density of 5 × 10^3^ cells/well in 96-well plates and allowed for overnight culture. Following confluence, the cells were incubated with various concentrations (0–100 µg/mL) of the sample for 48 h. After adding the MTT solution, plates were incubated for 3 h at 37 °C and 5% CO_2_. The microplate reader (TECAN, Männedorf, Switzerland) at 570 nm wavelength was used to quantify the colored formazan production which indicates cell viability.

### 4.8. Oil Red O Staining

3T3-L1 fibroblasts were differentiated into mature adipocytes according to Section 2.4. According to the previously established method, matured adipocytes were stained using Oil red O [69]. Briefly, after washing the cells with PBS twice, cells were fixed with 10% formalin for 1 h at room temperature. Subsequently, Oil Red O workable solution was added and stained for 10 min. The unbound stain was removed by rinsing with distilled water twice. Images of 3T3-L1 adipocytes were captured by an inverted microscope (DMI6000, Leica, Wetzlar, Germany). Quantification analysis was performed at 500 nm by extraction of the stain in isopropanol (Multiskan™ GO, Thermo Scientific™, Waltham, MA, USA).

### 4.9. TG Assay

A commercially available colorimetric TG assay kit (Biomax, Seoul, Republic of Korea) was used to assess cellular TG contents. After rinsing the matured adipocytes with PBS, they were resuspended with 5% NP-40. Samples were heated to 80–100 °C in the water bath till the solution became cloudy. Afterward, cell lysates were homogenized and centrifuged at top speed for 2 min and the TG content in the supernatants was quantified following the instructions provided by the manufacturer.

### 4.10. Lipolysis Assay

Based on the manufacturer’s instructions, a free glycerol reagent (F6428 Sigma-Aldrich St. Louis, MO, USA) was exploited to assess the free glycerol release by mature adipocytes at day 6.

### 4.11. Western Blot Analysis

The standard method was followed for Western blotting. Briefly, matured adipocytes at day 6 were lysed with ice-cold RIPA buffer supplemented with a protease inhibitor mixture (Concentration: 1 tablet for 20 mL RIPA buffer). The whole cell lysates were centrifuged at 12,000 rpm for 20 min at 4 °C and the supernatant was separated. The preparation of cytoplasmic and nuclear protein extractions was performed by exploiting a nuclear extraction kit (NE-PER™ Nuclear and Cytoplasmic Extraction Reagents, Thermo Scientific™, Rockford, IL, USA) based on the manufacturer’s instructions. After estimation of the protein amount by BCA assay, equal amounts (20 µg) of protein were mixed with 20% loading buffer allowed for separation by gel electrophoresis and transferred onto nitrocellulose membrane followed by blocking with 5% skim milk, and exposed to Western blot with primary anti-bodies (PPARγ, C/EBPα, SREBP-1, β-catenin, AMPK, p-AMPK, HSL, p-HSL, FAS, LPL, HO-1, Lamin B, β-actin) overnight at 4 °C. Followed by washing, the membranes were incubated with horseradish peroxidase-conjugated secondary antibody for 2 h at room temperature. A chemiluminescence ECL assay kit was used to detect the interest proteins and images were captured with a Davinch-Chemi Imager™ (CAS400SM, Core Bio, Seoul, Republic of Korea). ImageJ analysis software (ver.1.54g) was used for densitometry analysis.

### 4.12. Statistical Analysis 

The differences in the values were compared with one-way analysis of variance (ANOVA) followed by Duncan’s test using Sigma Plot ver.12.0 (Systat Software Inc., San Jose, CA, USA). Normal distribution of the variables and homogeneity of the variance was determined by the Shapiro–Wilk and Levene Median tests. All experiments were performed in triplicate with three technical replicates. Results are means ± SD (n = 3) and *p* < 0.05 indicates statistically significant differences among the groups.

## 5. Conclusions

**Figure 8:**
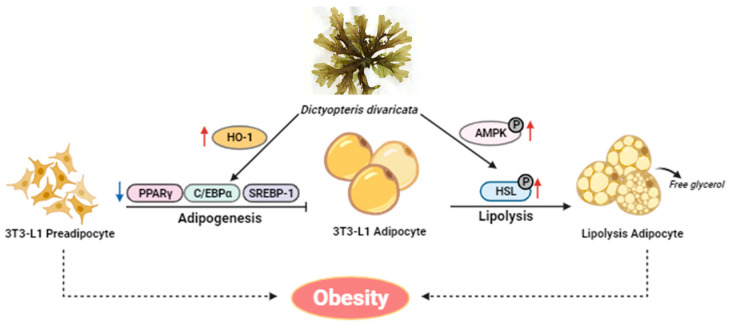
Proposed model of the anti-adipogenesis effects of the extract. *D. divaricata* inhibits adipogenesis by reducing adipocyte differentiation, and lipogenesis and promoting lipolysis in 3T3-L1 adipocytes.

*D. divaricata* showed an anti-adipogenic effect by inhibiting lipid accumulation, stimulating lipolysis, and ameliorating the expression of principal adipogenic transcription factors and lipid metabolism-related proteins. Moreover, ZnPP repressed the discovered outcomes of the *D. divaricata*-intervened anti-adipogenic effect. Taken together, the study results proposed that the HO-1-mediated molecular interventions might be a potential therapeutic scheme for obesity utilizing a natural nutraceutical like *D. divaricata*, nonetheless the in vivo effects of *D. divaricata* and its clinical importance remain to be clarified further (Figure 8).

## Figures and Tables

**Table 1:** Chemical composition of *Dictyopteris divaricata* extract.

R. Time	Compound Name	Molecular Formula	Peak Area (%)
17.253	(-)-Loliolide	C_11_H_16_O_3_	0.64
17.336	Tetradecanoic acid	C_14_H_28_O_2_	1.13
18.393	Neophytadiene	C_20_H_38_	0.73
19.826	9-Hexadecenoic acid	C_16_H_30_O_2_	1.01
19.949	Hexadecenoic acid, Z-11-	C_16_H_30_O_2_	0.38
20.147	n-Hexadecanoic acid	C_16_H_32_O_2_	11.46
22.376	2-Hexadecen-1-ol,3,7,11,15-tetramethyl	C_20_H_40_O	1.15
22.721	Linoelaidic acid	C_18_H_32_O_2_	2.20
22.846	7-Tetradecenal	C_14_H_26_O	14.79
23.153	Octadecanoic acid	C_18_H_36_O_2_	0.35
25.346	Arachidonic acid	C_20_H_32_O_2_	2.17
25.429	Doconexent	C_22_H_32_O_2_	1.27
28.624	Hexadecanoic acid, 2-hydroxy-1-(hydroxymethyl) ethyl ester	C_19_H_38_O_4_	1.02
31.399	9-Octadecenoic acid (Z)-, 2,3-dihydroxypropyl ester	C_21_H_40_O_4_	3.02
32.064	1,4-Benzenediol,2-(decahydro-5,5,8a-trimethyl-2-methylene-1-[1R-(1.alpha.,4a.beta.,8a.alpha.)]	C_21_H_30_O_2_	28.59
32.628	13-Docosenamide	C_22_H_43_NO	1.52
36.415	γ-Tocopherol	C_28_H_48_O_2_	3.22
37.466	Vitamin E	C_29_H_50_O_2_	1.53
39.948	Fucosterol	C_29_H_48_O	12.45

## Data Availability

The datasets of the current study are available from the corresponding author on reasonable request.
